# Gendered genital modifications in critical anthropology: from discourses on FGM/C to new technologies in the sex/gender system

**DOI:** 10.1038/s41443-022-00542-y

**Published:** 2022-03-04

**Authors:** Michela Fusaschi

**Affiliations:** University of Rome 3, Department of Political Science, Via G. Chiabrera, 199, 00145 Rome, Italy

**Keywords:** Health care, Quality of life, Human behaviour

## Abstract

Since the late 19th century, genital modifications (female and male) have been an important research subject in anthropology. According to a comparative and constructivist perspective, they were first interpreted as rites of passage, then as rites of institutions. In a complex dialogue with feminist movements, 20th-century scholars recognised that the cultural meanings of these modifications are multiple and changing in time and space. Conversely, according to WHO, since the 1950s, Female Genital Mutilation or Cutting (FGM/C) has been considered a form of Violence Against Women and Girls (VAWG). Interpreted as VAWG, FGM/C has progressively been isolated from its complementary male rite, selected for special condemnation, and banned. An order of discourse has been built by WHO and other international organisations. This article provides a genealogic deconstruction of the order of discourse lexicon, highlighting dislocations between anthropology and the human rights agenda. Today, genital modifications encompass FGM/C, male circumcision, clitoral reconstruction after FGM/C, gender reassignment surgery, and intersex and ‘cosmetic’ genital surgery. I propose to call these procedures Gendered Genital Modifications (GGMo). GGMo implicates public health, well-being, potential harm, sexuality, moral and social norms, gender empowerment, gender violence, and prohibitive and permissive policies and laws. The selective production of knowledge on FGM/C has reinforced the social and political polarisation between practices labelled as barbaric and others considered modern, accessible, and empowering. I suggest an anthropological interpretation for the socio-cultural meanings of health, sexuality, purity and beauty. I propose future interdisciplinary studies of how consent, bodily integrity and personal autonomy bear on concepts of agency and subjectivity in the sex/gender system.

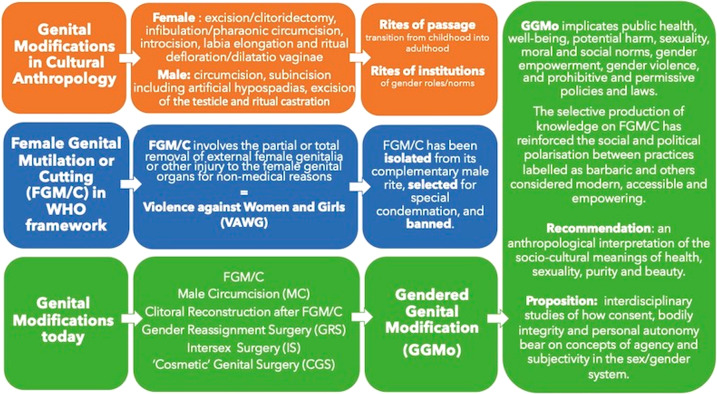

## Introduction

Since the late 19th century, the genital modification of females (through excision/clitoridectomy [1], infibulation [2]/pharaonic circumcision [3], introcision [4], labia elongation [5] and ritual defloration/dilatatio vaginae [6]) and males (through circumcision [7], subincision [8] including artificial hypospadias [9], excision of the testicle [10] and ritual castration [11]) has been an important research subject in cultural anthropology [12–14]. From a comparative and constructivist perspective, classical ethnography (i.e. the inductive method of long-term participant observation) considered these operations, which were conducted in many non-European contexts (e.g. in Africa, Oceania including Australia, and Southeast Asia including Thailand and Indonesia), to be initiation rites; specifically, they were seen as rites of passage [15], marking and facilitating the transition from childhood into adulthood [16, 17].

Early ethnographies provided accurate descriptions of how these rites (individual or collective) were carried out, highlighting ages, procedures, tools and ritual operators. According to this vision, genital modifications were also interpreted as bodily techniques [18] through which actors and societies reshaped the natural human figure to mark people’s social belonging. The irreversible cutting of the flesh was read as a ‘sign on the body’ and transformed the individual into an accepted member of certain religious and ethnic communities. Removing what is considered, from a social perspective, to be impure, unaesthetic and morally troubling helped individuals perceive themselves as approved, socially recognised women and men [19].

Since the mid-20th-century scholars recognised that the cultural meanings of genital modifications are multiple and changing in time and space. In a complex dialogue with feminist movements [20, 21], scholars explained the symbolic meanings of genital modifications: depending on the group, the context, and the type of modification, the meanings could include attempted preservation of a girl’s virginity prior to marriage, perception of increased fertility, increase or decrease in sexual pleasure, multiple levels of purity, protection against an alleged excess of sexual desire (whether in females or males), hygiene of the genital organs, elevation of status conferring respect required for marriageability and other gendered cultural values [22–27]. The anthropological perspective used to study the local cultural frame (i.e., emic vision) focused on the processes that society enacted on the bodies of individuals; this led to the acknowledgement that it is not only adulthood but also gender roles/norms that are socially instituted [28–30] by the cutting [31] or manipulation of genital tissues [32].

The World Health Organization (WHO) and the main UN agencies have defined female genital mutilation or cutting (FGM/C) as ‘all procedures that involve partial or total removal of the external female genitalia, or other injury to the female genital organs for non-medical reasons’ [33]. Because genital modification was not only salient to anthropologists, but also to political actors of various sorts, a Foucauldian ‘order of discourse’^1^ was built around FGM/C and this has become hegemonic in the global human rights system (see Appendix 1). Indeed, since the 1950s and through the ongoing enforcement of the human rights agenda, female genital modification has become the object of strong public and political debate.

From an anthropological perspective [34], as informed by the understandings of practising societies, female and male genital modification rituals were seen as symbolically linked [35], complementary practices within a *sex/gender system* (understood as the cultural processes by which sex, in a biological sense, is transformed into a gender as a social product^2^ [36]): *both* practices worked together to support, reinforce, and reproduce gendered relations in accordance with the prevailing norms of the local culture [37] (often local names are the same for the female and male rite). Moreover, these gender roles or norms were not necessarily organised around a hierarchical principle of female oppression and subordination; rather, prescriptive norms for men and women have varied widely across practising cultures, with power or status hierarchies often operating over age, for example, more than sex or gender per se. Such norms have undergone considerable transformation—and homogenisation—in certain societies as a consequence of the “colonial situation” [38, 39]; nevertheless, the value or status associated with male or female gender roles in contemporary settings continues to vary along multiple axes across different social domains (e.g. ethnicity), both within and between societies that practise genital modification (e.g. class). Bodily and sexual techniques are gendered incorporation processes and become a sphere of negotiation of social relationships between and inside genders and generations [40]. Thus, it is an oversimplification to assume that, wherever male and female ritual genital modifications occur, the female ritual is primarily oriented around socialising girls into a subordinate role [41].

Nevertheless, international actors opposed to female (but not male) genital modification have tended to ignore the male rites within practising societies while interpreting the female rites within the same societies in a highly reductive manner: as sex-discriminatory institutions of violence against women and girls (VAWG). Based on this interpretation, FGM/C began to be isolated: geographically, with a focus on African female genital modifications (ignoring Western-associated modifications, such as female ‘cosmetic’ labiaplasty or genital piercing); scientifically, with almost all subsequent research oriented around documenting harms as well as methods for elimination; politically, with opposition to FGM/C becoming a requirement for Western funding and favour; and anthropologically, becoming conceptually divorced from the parallel male rites occurring within the same *sex/gender systems*. The female rites also became re-defined (as barbaric mutilations), selected (placed into special typologies) and legally banned. This now so-called “female genital mutilation” or “FGM” became associated with irrationality, misogyny, primitiveness and serious harm.

At the same time, contemporary anthropological perspectives continue to stress and investigate the controversies (e.g. about preconceived Western ideas on genital modification and its origins, the importance of cultural relativism, the analysis of patriarchy as a Western inheritance, the recognition of the women’s social status, etc.) [42], projected cultural histories [43], impacts of colonialism [39], contested definitions [44] and terminologies [45], changes in paradigms [46], humanitarian reasons and moral economies [47], new postures (e.g. the modification as female empowerment) [48], and possible dialogues between different disciplines and movements [49]. Ignoring much of this research, the WHO has persisted in cordoning off and defining only non-Western-associated female-only genital modification as a grave human rights abuse, except when performed for ‘medical reasons’. Much of the academic literature, journalistic coverage of the topic, and international policy and law approaches to genital modification have taken their cue from the WHO.

However, a social change is now blurring this boundary. Questions are now increasingly raised about practices that have been biomedicalised in the West. In recent decades, various gendering surgeries have contributed to the biomedical construction of gender, often interpreted as new, technologically sophisticated, ‘liberal’ forms of genital modification. Gender reassignment surgery (GRS) and genital cosmetic surgery (GCS) emerged as ways to refashion one’s anatomy so that it is in line with one’s gendered and sexual preferences, desires [50] and identity needs. In the neoliberal framework, these surgeries are generally associated with health, modernity and empowerment. Further, intersex surgery (IS) [51, 52], primarily performed on infants and small children, is lauded by medical professionals as a modern ‘solution’ to sexually ambiguous genitalia; whereas, activists opposed non-consensual IS have identified it as intersex genital mutilation (IGM) [53]. Finally, male circumcision (MC), historically understood to be a ritual practice, has also undergone a biomedical transformation in the West: it is increasingly, albeit controversially, touted as a means of prophylaxis against infections and disease (so-called voluntary medical male circumcision or VMMC) [54]; meanwhile, activists who oppose the practice when performed on non-consenting individuals sometimes call it male genital mutilation (MGM).

Although these operations have all been constructed as being categorically distinct from FGM/C, they do share certain features with it that require consideration: they remove tissue from or otherwise modify a health vulva or penis and, when performed on infants or children, they raise ethical challenges concerning consent [55], autonomy [56, 57] and notions of bodily integrity [58]. As a way of resisting such uncomfortable comparisons, however, opposite imaginary constructs have emerged: ‘liberated bodies’ (Us) versus ‘victim bodies’ (Others). The deconstruction of these categories is necessary [59]. Going forward, it will be essential to know how, why and when genital modifications constitute a violation of a person or a form of gender oppression as well as when it becomes a gendered right. As new discourses [60] on the inviolability of one’s genitals arise and call into question state biopolitics, such questions will gain fundamental relevance in contemporary societies.

This article aims to provide a genealogic deconstruction of the genital modification lexicon, focusing on fractures and dislocations between anthropological perspectives and global human rights policies. Regarding female genital modifications, I argue that the ‘FGM/C’ order of discourse has produced a real ‘heterogony of ends’ (a process whereby original motivational intentions become modified through a chain of events). I also highlight the uses and abuses of anthropological vocabulary. Finally, I suggest a new interdisciplinary method based on gendered ethnography (as a political critique) to analyse all genital modification practices. This method allows for providing due attention to the intersubjectivity of social actors and researchers as well as management of gender-power-related tensions that could arise in the field; it aims to produce knowledge that may contribute to scientific and non-scientific fields [61]. Paraphrasing Butler, ethnographic fieldwork is the way to yield to another’s experience, dignify the words of both social actors and the researchers involved, and to grasp the meaning of life practices in a constant intercultural dialogue [62].

## The genealogy of FGM/C’s order of discourse

Since colonial times, genital modification has been the subject of politics and policies. As an illustration of this, we will remember the colonial dispute between British Christian missionaries and the Kikuyu, a Kenyan ethnic group, regarding the colonial ban on female ‘circumcision’ [63]. On the one hand, this colonial decision was met with tumult and a major boycott of mission schools and churches by members of the Kikuyu; on the other hand, it served as a cause to rally African support for Kikuyu political leaders. One of the consequences was the birth of independent schools and churches as a form of autonomy from British domination [64].

Since the end of the Second World War, the issue of female genital modification has become a problem in the global North; this is because it fits into the discourse of human rights and the birth of major international organisations as outlined in the vision of the Universal Declaration of 1948 and in the Geneva Conference of the Society for the Protection of Children (1948) [65].

One year earlier, in 1947, the Executive Board of the American Anthropological Association (AAA) prepared a statement on human rights and submitted it to the UN Commission on Human Rights. Briefly, the AAA critiqued what they saw as an ethnocentric position (i.e., the belief in the superiority of one’s own culture or ethnic group) endorsed by the UN, instead proposing to centre cultural diversity through the concept of cultural relativism: roughly, the belief that certain values, instantiated in practices, may be relative to specific cultures and should therefore not be denigrated simply on the grounds that they do not conform to one’s own cultural standards.

The AAA’s statement, which was based on this notion of cultural relativism and the belief that no substantive declaration of rights could be meaningfully applied to all human beings irrespective of their cultural context [66], marked a hiatus of a productive dialogue between anthropology and international conventions concerning human rights. We can call this period ‘the birth of the order of discourse,’ [67] and it can also be understood from the attitude of the UN agencies and as defined in several conferences (see Appendix 1).

In 1958, the UN Economic and Social Council asked the WHO ‘to undertake an inquiry into the persistence of these practices (meaning female genital modifications) and into measures adopted or planned to stop the ritual operations’ [68]. In 1959, the WHO answered that ‘the ritual practices in question resulting from social and cultural conceptions are not within WHO’s jurisdiction’ [69]. In other words, at first, the WHO had declined the invitation. Yet, in its resolution 821 II (XXXII), which was adopted in 1961, the UN Economic and Social Council again invited the WHO to study at least the medical aspects of the female-affecting operations based on custom. For almost a decade (the 1960s-70s), the WHO and other international organisations, such as UNICEF, saw the FGM/C as a cultural problem (and not a public health one); they assumed that it needed to be analysed (e.g., data collection) and resolved by the local politics of the countries involved. As a result, probably, for this reason, WHO did not respond to the UN Economic and Social Council.

But it is in this same period, from a descriptive and interpretative view of cultural complexity (as evidenced by ethnography), we saw a dislocation of meaning within the notions of culture, ritual operations, and tradition, which are the basis of anthropological knowledge. They acquired political value and became descriptive notions in the sense of classification: Us and the Others. These anthropological concepts were considered using an ahistorical and essentialist perspective: ‘seemingly *universal* essentialist generalisations about “all women” are replaced by *culture-specific* essentialist generalisations that depend on totalising categories such as “Western culture,” “Non-western cultures,” “Western women,” “Third World women”’ [70] and so on. Some cultural practices were conceptualised as harmful only for specific categories of human beings: women and girls (i.e. minorities to be protected). Despite the allure ‘of such grand metanarratives, gender essentialism produces a theory that effaces the differences between women’ [71] both within and between cultural contexts or societies. In this victimisation rhetoric only women and girls who live in contexts conceptualised as barbaric (e.g., Africa), where operations of this kind take place for cultural reasons, need to be saved.

To international organisations, anthropology seemed to be the most appropriate discipline to draw on, because it studies cultural diversity (the Others). The nascent theories of social change (i.e., consideration of human relations as interactions shaped over time) and cultural pluralism were not functional for the purpose of political discourse. The only targeted concept was the culture (understood in a primitive and essentialist significance, as barbaric tradition), which was considered the basis of violence against women. In the anthropological sense, the culture is a symbolic system integrated, shared and dynamic, something we acquire during all our lives.

It is no coincidence that, in addition to genital practices, other cultural practices (e.g., child marriage, female infanticide, menstrual stigma and nutritional practices) were considered by UN agencies as a type of social pressure through which men oppress women via patriarchy. This is a concept that feminist anthropology has intensely criticised when it is assumed in an ahistorical sense; it is believed that all men (men as a class or category) prevent women from freely expressing themselves or (otherwise) exercising agency, and, consequently, men become the silencers of women. In this rhetoric, women in the Global South emerge as victims to be saved because they are represented as uneducated, unlettered, custom-bound, oppressed, domesticated and forever victimised [72]. The body is a place where men reinforce their strength (note: also through male circumcision) by weakening the female body with ‘harmful traditional practices’ [73]. The harm is the expression of violence, which is carried out directly on the body, sexuality and, finally, on health; this is especially true for African women, a category that is an abstraction/invention from the anthropological point of view. African women’s bodies will be interpreted as symbols of oppression at the hand of barbaric and uncivilised practices, and their sexuality will be compromised. (see Box 1).

Based upon this framework, the operations that had been defined in 1967 as ‘male genital mutilation’ by anthropologist George Peter Murdock [74] were expelled from the order of discourse because they were not seen as instances of violence against women nor as adversely affecting health (that is, setting aside ‘botched’ operations; male sexual health was thus conceptualised in a heteronormative and reductive sense as the capacity for erection and ejaculation/orgasm, so as to potentially impregnate a female partner; the sexual sensations and affordances of the foreskin itself are erased on this conception). At the same time, the female operations—still sometimes called ‘female circumcision’ but increasingly ‘FGM’ – were isolated geographically (imagined to be quintessentially “African”), and defined and classified by the WHO into four typologies (roughly: type 1 – modifications of the clitoris; type 2 – modifications of the labia; type 3 – infibulation; type 4 – miscellaneous); meanwhile ritual defloration and ritual dilatation (i.e., widening of the vaginal orifice) ceased to be matters of concern. This order of discourse was consolidated in the 1970s; these were the years characterised by second-wave feminism and, among other things, the idea of global solidarity of women. Specifically, the task of women living in the North of the world was to save women in the South. The body was the pivotal point of the discussion because it became a politically oriented project of self-determination. The slogan ‘personal is political’ worked well in the discourse to denounce female genital modifications.

The years leading on from the 1970s and into the 1980s became a turning point for the consolidation process of the formula ‘Female Genital Mutilation’ in a political sense. On the academic front, Rose Hayes, for the first time, used the expression ‘female genital mutilation’ to describe the cultural/functional/structural complexity of infibulation in the Sudan [75]. That article remains one of the most interesting interpretative texts of the period, as it is based on rich fieldwork in which anthropological instruments were used to clarify that the ‘mutilation’ was only part of a more comprehensive ritual apparatus. Regarding genital modifications, analysis showed that the deconstruction of the physical body involves a ‘construction’ of the symbolic one within the sex/gender system (the relationships/hierarchies both of gender, male and female; and of generations, between older and younger women). Concepts like multi-gender positioning (i.e., taking multiple or different gendered positions during life) [76] and subjectivity (i.e., a specific cultural and historical consciousness existing at the individual and collective level)^3^ [77, 78], had started to develop in feminist ethnography but did not seem entirely useful for political purposes of the female genital modification discourse. However, the expression ‘FGM’, which reflects the assumptions of the international agenda and framework for elimination of the female rites, provided an explicit condemnation and denunciation of what was conceptualised as a ‘harmful traditional practice’ where traditional became synonymous of violent and savage. It also provided an implicit ‘condemnation’ of the anthropological discipline that advanced alternative or neutral terminologies (e.g., circumcision/circumcision of girls, female genital cutting, female genital modification^4^ female genital alteration, female genital surgery and local terms) [79].

The adoption of the acronym ‘FGM’ took place in the political arena through the contributions of one of the most influential but ambiguous figures [80] of the period: Franziska Porges Hosken, also known as Fran Hosken. Hosken, born to a Jewish family in Vienna (her father was a physician) and later immigrating to the United States at the age of 18 in 1938, was a designer (with a degree in architecture), writer/journalist, photographer and, later, adviser to WHO and representative of Western feminism [81]. She didn’t coin the term ‘FGM’ - as often claimed - but she did use the term to politicise the issue in 1976 [82] and then in 1979 in the context of the first large-scale survey on the subject. *The Hosken Report: Genital and Sexual Mutilation of Females*. Hosken presented on this survey at the WHO’s *Seminar on traditional practices affecting the health of women and children* in Khartoum. This report was used as a textbook for years. Hosken herself condemned both female genitals cutting and the very discipline of anthropology because, at the time, most anthropologists were men and, according to her, ‘naturally’ embodied power, and domination. It was certainly not a coincidence that the topic of ‘FGM’ became a ‘separate subject’ of sorts studied primarily by (and for) women researchers and feminists.

During the 3rd UN World Conference on Women in Nairobi (1985), the concept of *gender mainstreaming* was introduced. In principle, gender mainstreaming (as a strategy for promoting gender equality) and *empowerment* (the process by which women gain power and control over their own lives and acquire the ability to make strategic choices) should have been positive concepts; however, both concepts became the passe-partout of global hegemonic differentialist politics. Some scholars illuminated how, because they represent a Western idea projected onto the Other’s social relations and sex/gender systems, these definitions are ambiguous and polymorphic. For example, the concept of gender mainstreaming refers to many different things (e.g., access to technology or gender equality in parliament). Further, the idea of empowerment implies that an external authority gives power to women, and those women are often wealthy, with their own agendas and preconceptions, which diminishes poor women’s agency [83].

Through these and other developments, the FGM vocabulary became solidified despite local mistrust, conflicts of interest, and other controversies, seen in large part as a medical (i.e., women’s health) issue until the 1990s. The order of discourse then became a hegemonic act of humanitarian reason, which indicates that the introduction and promotion of moral sentiments into human affairs become the essential element of contemporary local and global policies [84]. This posture, which is compassionate and repressive at the same time, authorised the criminalisation of FGM/C. Consider, for example, the 4th World Conference on Women in Beijing (1995) (see Appendix 1) [85]. The 1990s were years of paradigmatic change ‘from health to human rights’ [46], and then a combination of the two [86], and saw significant criticism about politics of interference by the post- and de-colonial feminist movements.^5^ FGM/C became the focus of global debates on VAWG, gender-based violence (GBV) and reproductive rights. Despite the diffidence exhibited by some feminist anthropologists [88, 89], FGM’s definition (i.e., a formula that excludes and homogenises operations that do not include an actual cut; see Box 2) is followed by a politics of naming and classification [87].

As a result, these ‘imperfect glossaries’ based on ahistorical notions (e.g., notions of ‘culture’, ‘tradition’, ‘potential victims’, ‘victims at risk’, ‘harmful traditional practices’, ‘social pressure’ and ‘for non-therapeutic purposes’) have been locally incorporated and become part of the vernacular. The process of vernacularisation ‘is one of appropriation and translation. Human rights ideas and feminist ideas are appropriated by national elites and middle-level social activists and translated into local terms’ [90]. As some studies have shown, the real effects of such appropriation/translation in the field can be profound [91] (e.g. the impact of African women reformist elites and the developmentalist colonial state on the experience of girlhood in Nigeria, as demonstrated by George Abosede [92]; or the problematic incorporation of gender mainstreaming in the humanitarian discourse on *gukuna*, a Rwandan genital manipulation [93]) (see Box 2 for discussion).

Despite their shortcomings, these glossaries became the frame of reference, not only for many academics and medical researchers, but also within international organisations, NGOs, local legislation and governments (see Box 3).

This vocabulary was also legitimised across interactions between moral economies and the seduction of quantification [94] and allowed FGM to be conclusively positioned within the framework of its criminalisation. Contemporary moral economies characterise a specific historical moment and, sometimes, a particular group (e.g., minorities, such as migrants, refugees, women). By analysing the moral economy, we consent to understanding ‘the production, dissemination, circulation, and use of emotions and values, norms and obligations in the social space’ [95]. The representation of suffering (e.g., through ‘shocking’ or graphic images and narratives, whereby the most extreme, non-representative outcomes are used to illustrate, or stand in for, the entire class of procedures/effects) has become increasingly commonplace in the public sphere, including on social media and in the political arena, and it has defined the strategies of (bio)power to justify action. Utilising the imperfect vocabulary, FGM has been defined historically as a ‘pressure norm’ (i.e., a violation of individual and collective rights of women in the global South), resulting in its condemnation. On the other hand, for example, aesthetic surgery has been framed as a ‘social norm’ and legitimised as an expression of freedom of choice among Western women.

Consider an example of such conflicting interpretations. Eugenia Kaw argues in her ethnography that, in the US, the demand for cosmetic surgery to be performed on Asian American women’s ‘racial traits’ is intensified by stigma experienced by racialised minorities in a white-dominated society [96]. According to her interpretation, Western culture has progressively produced the idea that Asian facial features are synonymous with an emotionless and pallid expression. Korean women feel inadequate and have internalised the ‘self-racism subtext,’ then request surgery to distance themselves from these negative features and avoid being viewed as passive subjects lacking in sociability. In this framework, where cosmetic surgery is presented as a form of empowerment and freedom of choice, cosmetic surgeons become the ‘producers of the norm’ and contribute to the process of ‘acceptance’ and social homogenisation. The choice of surgery, more than a transformation (i.e., ‘beautification’ in the aesthetic surgery vocabulary), is a process of gender normalisation conforming to Western/white definitions of femininity and beauty.

At the same time, cosmetic surgery in the neoliberal era seems to provide social and economic mobility as a synonym for success, which explains its contemporary popularisation and normalisation. This example illuminates the ‘paradox of choice’ (i.e., circumstances that leave social actors, especially women, without real options, as shown by Kathryn Morgan) [97]. Morgan identifies three paradoxes: the choice of conformity (i.e., replacement of minority identity with white conformity), the liberation into colonisation (i.e., voluntary mutilation to create a new accepted body; the body is seen as a raw material to be shaped to fit external standard—the white one), coerced voluntariness and the technological imperative (i.e., social pressure, such as that experienced through exposure to social media and advertising, to achieve perfection of femininity through technology). This is a useful concept for understanding also whether gendered surgery is liberating or coercive.

Returning to FGM/C, according to UNICEF, ‘more than 200 million girls and women alive today have been subjected to the practice, according to data from 30 countries where population data exist’ [98]; this prevalence estimate forms the basis of the discourse that views FGM as a form of GBV. According to Merry, such quantification is seductive because it offers numerical information to describe, compare and rank different things (e.g., jobs, schools, aesthetic surgery, gender violence). This consolidated ‘indicator culture’ shapes neoliberal governance on a local and global level. On the one hand, the indicators draw on subjective data about social phenomena, quantify it, and present it as true and objective. On the other hand, the numbers silence social actors, such as the feminist movements of the Global South. The indicators simplify complex local and social dynamics. The quantification can be risky because what is calculated and represented also influences the common sense of what needs to change and how to do it [94].

Because the FGM discourse has been incorporated locally, moral economies and quantitative measurements produce social effects that must be critically investigated; it is especially important that we investigate their impact on the process of subjection [99] in the double sense of ‘becoming subordinated’ in the sex/gender system as well as ‘becoming a subject’ (also resistance). Both aspects of the process of subjection should be analysed via interdisciplinary and comparative fieldwork (with other/new forms of genital modifications) to help intercultural dialogue.

Box 1 The challenge of bodies and sexualities gendered conceptualisations In recent decades, some scholars have stressed how the Western conceptualisations of bodies and sexuality are mainly connected to the dichotomy of male and female. Gender binarism opposes men to women, where the first one is presented as a superior and the second as an oppressed category. As ethnography has demonstrated since the 1930s, this framework is particularly problematic to many cultures. More recently, Oyeronke Oyěwùmi [131] and Sylvia Tamale [132] explained how African realities, bodies and sexualities (even those of Arab homosexuals, as outlined by Joseph Massad [133]) have been interpreted based on universalistic and essentialised Western categories. Due to the incommensurability of local categories and social institutions, this posture produces distortions, confusions in language and, often, a total incomprehension. The gendered categories also demand a complete rethinking. It is important to consider historical Western hegemony, which is defined as imperialist by some scholars. A genealogy of the ‘inventions of traditions’ (i.e., the intricate colonial and neo-colonial ideologies that social actors embody) becomes imperative. Moreover, it is crucial to reconstruct the rhetoric related to modernity and agency, childhood, girlhood, family, bodies and sexualities.

On the one hand, this reconsideration permits us to recognise the complex structures within which sexuality is elaborated in its pluralist articulations (i.e., experiences, identities, and the relationships between power and desire). On the other hand, it allows an understanding of how the experiences of gender and sexualities are shaped and re-defined by issues, such as neo-colonialism, neoliberal globalisation, (bio)powers, social stratification, and religion. Bodies and sexualities offer ‘unending lessons about pleasure, creativity, subversion, violence, oppression and living’ [134] and the possibility for researchers to deconstruct neoliberal vocabulary around gendered categories in relationship with other actors, such as movements, activisms, and non-governmental organisations (NGOs).

Box 2 Type IV: the dislocation of labia elongation/stretching *Eliminating female genital mutilation* is an interagency statement of the United Nations (WHO, 2008) [135]. This document classifies FGM/C into four types (see the main text for a brief summary) reflecting certain adjustments to accommodate concerns and shortcomings of previous declarations going back to 1995. Under Type IV (miscellaneous or unclassified), we can compare the 1995 definition with the 2008 update: as follows:

Type IV in 1995: pricking, piercing or incising of the clitoris and/or labia; stretching of the clitoris and/or labia; cauterisation by burning of the clitoris and surrounding tissue; scraping of tissue surrounding the vaginal orifice (angurya cuts) or cutting of the vagina (gishiri cuts); introduction of corrosive substances or herbs into the vagina to cause bleeding or for the purpose of tightening or narrowing it; and any other procedure that falls under the broad definition of female genital mutilation.

Type IV in 2008: All other harmful procedures to the female genitalia for non-medical purposes; for example, pricking, piercing, incising, scraping and cauterisation.

We can see that the WHO removed labia elongation/stretching from the classification of Type IV. However, in Appendix 2 of the same interagency statement, we find a *Note on the classification of female genital mutilation*. In the paragraph titled *Stretching*, we can read: “Stretching or elongation of the clitoris and/or labia minora often referred to as elongation, has been documented in some areas, especially in southern Africa. (…) Labial stretching might be defined as a form of female genital mutilation because it is a social convention, and hence there is social pressure on young girls to modify their genitalia, and because it creates permanent genital changes” (p. 27).

This statement is a tool, and this dislocation could generate some confusion especially in the humanitarian field. For example, in the last two decades, I analysed the Rwandan ritual of gukuna (labia elongation through mutual female massage). In the local sexual cosmology, this modification with the kunyaza (male sexual technique of genital stimulation) is expected to increase fertility and sexual pleasure. When I started my fieldwork, in the late 1990s, the gukuna was conceptualised as a secret of “liquid female sexuality” (production of abundant vaginal secretions before and during sexual intercourse as the guarantee of a good marriage) [32]. In the mid-2000s, new subjectivities started to emerge pro or against gukuna. Furthermore, the movie ‘Sacred Water’ (2016) and some newspaper articles revealed the gukuna to the broader public, as a mystery of female ejaculation.

On the one hand, the anthropological interpretation of *gukuna* showed some agency (female pleasure) and challenged victimising assumptions and hegemonic representations of both ‘FGM’ and ‘the African Woman’. On the other hand, Appendix 2 is locally used by some NGOs and anti-FGM movements to continue to characterize *gukuna* as ‘FGM’ and so something to be eradicated, even if it is no longer explicitly mentioned under Type IV of the WHO typology.

Box 3 An unusual concept of culture in an official Italian document In 2009, the Italian Ministry for Equal Opportunities financed the Quantitative and Qualitative Evaluation of the phenomenon of FGM in Italy; this was done at Istituto Piepoli - Marketing and Opinion, with funds allocated by criminal law 7/2006 on FGM. This research, known as the Piepoli report, has several limitations, which are also demonstrated by the European Institute for Gender Equalities (EIGE). The overall quantitative methodology is not clearly explained, particularly regarding data and quantification, and some conceptualisations represent a model of archaic and racist vocabulary. For example, in the section titled *African women in Italy: The Culture*, we can read:

‘The morality of African women requests an attitude of introversion. (…) African women have been socialised in cultures characterised by solid social networks with ritual components (…); they differ profoundly from other immigrant realities (…). The facial features, the tattoos, the scarifications, the language and the FGM permanently marks the belonging to a group: to the women’s group, to the African Women’s Group, to the group of this specific area and this female-specific tradition’ (p. 31).

The European Institute for Gender Equality (EIGE) has criticised the entire methodology of this study [136]. It’s important to stress that for years, this report was the official document in public awareness initiatives in Italian health care centres, voluntary organisations and NGOs, creating many comprehension problems and tensions between operators and minorities.

I was invited to many conferences in many Italian municipalities to discuss these limitations and to deconstruct this lexicon [72]. My critical analysis focused on the messages that this report conveys both “humanitarian moral”, the racism and ‘differentialist neo-sexism’ (the perception of migrant women’s bodies in the Italian society as subordinate and victims’ subjects) [137].

## The new technologies of gender

From an anthropological point of view and out of FGM/C’s order of discourse, I suggest that hymenoplasty [100, 101], labia elongation [93], intimate cosmetic surgery (male [102] and female [103]), clitoral reconstruction [104], male circumcision [105, 106], gender reassignment surgery (GRS) and operations on intersex new-borns [51] should be understood as technologies of gender [107] under a local hierarchical sex/gender system. The distinguishing feature of these practices is that they are concentrated on the sexual anatomy and require ‘surgery’ [108]. GRS, which is widely considered to be an enhancement of trans people’s rights, is increasingly being contested where it is required to gain one’s desired administrative identity. Furthermore, IS and male circumcision are being denounced as mutilation [109] by secular activist movements that defend LGBTQ + rights. Clitoral reconstruction [110], which is sold as surgical ‘repair’ of FGM/C is now offered in several countries, with growing criticism of the practice from various corners.

Conversely, despite multiple laws and prevention projects [111] aimed at ‘eradicating’ it, so-called FGM/C persists in part because of its increasing biomedicalisation in various countries. The FGM/C biomedicalisation, sometimes in local context is perceived as dissolving health risks. Moreover, along with hymenoplasty, which some women seek to restore a cultural marker of supposed virginity, the appeal of Cosmetic Genital Surgery (CGS) is growing rapidly, even for girls under 17 years old.^6^ [112, 113]. These latter practices are being presented in the media and in public discourse as appealing fashion choices (e.g., by being referred to as ‘designer vagina’ [114], ‘barbiplasty’, ‘vaginal rejuvenation’ [115] and the ‘enhancing of sexual life’), and they do not raise public concern [116]. The hegemonic scientific approach (which takes for granted the opposition of customary versus modern, oppression versus freedom, and traditional practice versus surgery) thus produces knowledge by separating these practices [117] and is inadequate in answering new socially relevant questions about freedom or coercion, desire and oppression, etc.

In the realm of development projects and immigration policies [91], some studies have questioned the isolation of FGM/C, arguing that it should be compared to other forms of genital modification, such as male circumcision. Anthropological fieldwork has rarely addressed the recent shifts in meaning regarding male circumcision in the West [34], particularly within the framework of the ‘multicultural dilemma, as a “problem” of minority groups that wish to maintain “ritual” practices for “religious” reasons, which can, subsequently, only be made acceptable throughout medical interventions’ [118]. Consistent with this interpretation, in recent decades, the global public health agenda reframed the cutting of the foreskin as beneficial for the prevention of HIV [119]. Thus, male circumcision is now promoted as a health practice in the countries of the global South, especially in Africa, including contexts where circumcision was not previously practised. Simultaneously, male circumcision is being contested [120] in the global North as a form of gender violence [121].

These changes have been studied from biomedical, juridical and bioethical viewpoints; [122] however, they have not been studied enough from a gendered ethnographic-based perspective, which could clarify how cultural meanings of the body, impurity, sexuality and religion are being reframed. Today, a critical analysis of policies and legislation as well as symbolic, social and biomedical dimensions are fundamental. In other words, a study of the ‘mindful body’ [123], (defined by Nancy Scheper-Hughes and Margaret Lock as an *in fieri* construction in the interlacement between dynamics of production, reproduction and cultural reinvention) is increasingly necessary.

Medical anthropology suggests three perspectives for understanding the mindful body: the *individual body* (lived experience), the *social body* (as symbol), and the *body politic* (referring to the regulation, surveillance, and control of reproduction and sexuality in work, etc.). The selective legislation regarding genital modification (e.g., medically unnecessary female, but not male or intersex, child genital cutting being prohibited) places a higher value on the political body (e.g., in reproducing gendered norms around victimhood) than the other two dimensions. For these reasons, it is crucial to rebalance the approach, redistributing the importance of the three dimensions. It is fundamental to ethnographically examine the individual and social dimensions of genitalia and to historically situate the prejudicial production of the political dimension.

It is also required that we investigate how—through the biomedicalisation of genital modification—the social dimension articulates with the individual one. The first dimension focuses on issues regarding gender binarism, purity and health, while the second focuses on the lived experience of harm/violence and/or empowerment as outcomes of the procedures. It is crucial to evaluate harmfulness and agency without neglecting the subjectivity of the people who undergo gendered genital modifications (GGMo).

Furthermore, there is a lack of ethnography that addresses the biomedical setting of male cosmetic surgery, genital piercing and IS. It is important to study how cultural assumptions regarding genitals, beauty, sexuality, ageing, the inviolability of the body and gender norms are embedded in scientific knowledge and clinical work surrounding genital surgery in different settings. Understanding the point of view of professionals and the ‘delegated biopolitics’ [124] is a new challenge; this concept of biopolitics encompasses three dimensions of delegation: delegation to the patients (who must consider options and consent to the consequences of their decisions); delegation to the healthcare professionals (who, during one-on-one consultations, are obliged to test the strength and steadiness of their patients’ desires and how informed they are); and delegation to the professionals in biology and medicine (who must implement their instruments of self-control in ethics committees and hospital protocols). The analysis of this triple delegation represents a possible key to understanding the different types of power that could be exercised over individuals, along with their needs or desires and the role of institutions in the choices of individuals.

In this context, the negotiation of consent and mobilisation of cultural meanings pertaining to health, beauty and sexual enhancement are presented as new challenges. The progressive medicalisation of FGM/C in certain contexts and the rise of other genital surgeries are issues that require attention. Furthermore, the literature that addresses intimate cosmetic surgery under neoliberalism does not adequately account for new gender surgery technologies.

By theoretically discussing consent, violence and harm, philosophical and bioethical literature [125], along with gender, political and legal studies [108], some scholars have tried to account for the multiple forms of genital surgery [126]. A problem with the existing literature, however, is the relative lack of attention to subjectivity and gendered multi-positionality, which are central in the anthropological debate. However, these notions become fundamental when rearticulating the relationship between universalism and cultural relativism.^7^ The theoretical discussion of violence must consider the historicity and knowledge of the different cosmological and symbolic meanings concerning each practice in its local (and trans-local) context.

The anthropologist Veena Das argued that the concept of violence is extremely unstable. She proposed that, instead of policing the definition of violence, we accept its instability as crucial to the understanding of how the reality of violence includes its virtuality and has the potential to make and undo social worlds [128]. Also, the sex/gender system is crucial for understanding what connects local and global visions and their historicity.

Furthermore, the literature has not addressed gender binarism as the intersecting and distinguishing feature of these practices. The emerging movements (e.g., intactivism)^8^ [129] reveal the idea of the ‘inviolability’ of one’s genitals and the dynamics of re-naturalisation of the body; consequently, a denunciation of male modification as a form of gender violence appears. The contestation of ‘forced’ gender binarism (as in intersex surgeries) could foster unprecedented positions of pro-body integrity. Comparative and fieldwork research that interrogates this apparent paradox is increasingly necessary. As it stands, there is yet insufficient historical and ethnographic inquiries into the new anti-circumcision and intactivist movements or secularist, religious, and state-new prohibitionist policies against IS [130].

## Conclusion

GGMo is an increasingly popular biomedical set of practices that entails compelling issues, such as public health, well-being and potential harm; sexuality, virtue, and moral and social norms; gender empowerment and, conversely, gender violence; and prohibitive and permissive policies and laws. GGMo can be delivered through various methods, following different biomedical settings and different cosmologies. However, all forms of genital surgery entail issues surrounding gender enhancements and agency. Today, GGMo encompasses male circumcision, FGM/C, clitoral reconstruction after FGM/C, GRS, IS on newborns and CGS. Until now, the production of knowledge and public discourse has kept FGM/C separate from other forms of genital surgery. Furthermore, discussions of FGM/C have been built on a performative order of discourse, which is now incorporated by social actors and public opinion. Because it is considered a form of violence against women and girls, FGM/C’s social and cultural dimensions should receive increased scientific attention.

In contrast, CGS, which can also involve children, has thus far remained unexplored, particularly concerning its potential harmfulness. We need to ask how these practices are selectively condemned and commended by the different actors in the field and which social and moral stakes are encompassed. Moreover, the theoretical stance that considers female GCS as the unracialized mirror of FGM/C has developed without any ethnographic description of how aesthetic genital surgery is morally legitimated, culturally sought, socially organised and delivered.

The selective production of knowledge has reinforced the social and political polarisation between practices labelled as customary and barbaric and others considered to be modern, free and empowering. Important matters of racism, neo-colonialism, ethnocentrism and victimisation have arisen from this divide. Moreover, the laws and public policies criminalising FGM/C have resulted in the clandestine delivery of these practices.

Until now, mutilation was the name given to a specific form of genital surgery cast as non-therapeutic and targeting only women and girls. Recently, the characteristic of harmfulness has been increasingly attributed to those practices that pertain to biomedicine, which include IS, GRS and male circumcision. New social movements have emerged (e.g., intactivists, post- and de-colonial feminists), and they raise relevant questions about consent, the role of the state and biomedicine in preventing harm, and the moral threshold regarding the modifiability or inviolability of the gendered body. Understanding this change entails considering questions too often left to specialists in medicine and psychiatry (e.g., the question of what is therapeutic or harmful in genital surgery). An anthropological and ethnographic approach that can account for the socio-cultural meanings of health, sexuality, purity and beauty is fundamental. Nevertheless, we need an integrated and collaborative anthropological analysis of how, in this realm, consent, integrity and autonomy articulate with the concepts of agency and subjectivity in the sex/gender system.

## Supplementary information

Appendix 1

## Footnotes

1 As defined by Michel Foucault, the *discourse* relates to ways of organising knowledge and meaning in a social system historically, via modes of subjectivity and power relations. The exertion of power shapes concepts and categories for legitimating knowledge into a discursive order; the discourse fixes meanings in a way that is favourable to the dominant political system and the logic that underlies its production. In this sense, the discourse normalises and homogenises, including through interventions into the bodies and subjectivities of those it dominates (incorporation). Because it can come to be interpreted as objective, stable and universal, this process constructs a system of control (biopower) and involves various kinds of bodily discipline (e.g., in genital modifications and in the sex/gender system).

2 The anthropologist Gayle Rubin defines the sex/gender system as ‘the set of arrangements by which a society transforms biological sexuality into products of human activity, and in which these transformed sexual needs are satisfied’ (1975). Because sexuality is unrelated to anatomical genitalia, biology is not a problem; rather, structures of social organisation (such as inequalities of status and power) are problematic. Kinship systems and child education are, among other things, observable and empirical forms of local sex/gender systems. The oppression and subordination of women can be interpreted as a product of the relationships (heterosexual) by which sex and gender are hierarchically organised and produced. Sex/gender system is a fundamental concept to understand how sexuality is categorised and how gender is produced socially. It is also a tool to deconstruct the trend toward biological determinism and for analysing different forms of the family and their transformations over time and space.

3 Subjectivity refers to a specific cultural and historical consciousness at both the individual and collective level. At the individual level, actors are always at least partially ‘knowing subjects’ in that they have some degree of reflexivity about themselves and their desires. At the collective level is the collective sensibility of socially interrelated actors. Consciousness, in this sense, is always part of people’s subjectivities and part of the public culture.

4 Since 1999, when I started fieldwork in Italy and Rwanda, I substituted the term ‘mutilation’ with ‘modification’ to build a neutral, prejudice-free space with social actors. Using the word ‘modification’ permitted me to refuse any generalisation and simplification of talking with women at an egalitarian level. This methodological cultural relativism, which was not a form of justification, taught me that reversible or permanent body modifications are universal in the sex/gender system.

5 See, e.g. the *Dafay Network*, ‘a network of actors in the field of research, artivism, & political and activist action, engaged in the decolonization of knowledges, discourses and intervention practices on Female genital cutting in Europe and the global North’ https://baadon.com/en/workshops/. See also Fuambai S. Ahmadu’s website, http://www.fuambaisiaahmadu.com. Fuambai S. Ahmadu is both a professional anthropologist and an initiated member of the Bondo society of Sierra Leone. She says she was circumcised, not mutilated, and she chose to undergo the procedure. [87].

6 The International Society of Aesthetic Plastic Surgery provides the only statistics about the number and type of aesthetic procedures performed on a global scale. https://www.isaps.org/wp-content/uploads/2019/12/ISAPS-Global-Survey-Results-2018-new.pdf

7 Marie Benedicte Dembour, anthropologist of law, has proposed the ‘theory of the pendulum’. She argues that universalism and relativism are usually presented as two opposite and incompatible moral (or epistemological) positions regarding human rights. The position proposed by the author is unstable and encompasses both the universalist and the relativist stances. It is not a middle position but an in-between position that makes sense of the fact that a social actor should be drawn into a pendulum motion. [127].

8 For example, the French association *Droit au corps* that aims ‘to promote the abandonment of all forms of sexual mutilation - female, male, transgender and intersex: excision, circumcision or other - i.e. any modification of a sexual organ practised on an individual without his or her free and informed consent, and without medical necessity.’ (https://www.droitaucorps.com). Recently also some newspapers covered this item, see The Guardian, https://www.theguardian.com/society/2019/jul/21/foreskin-reclaimers-the-intactivists-fighting-infant-male-circumcision.
