# Correction: Gendered genital modifications in critical anthropology: from discourses on FGM/C to new technologies in the sex/gender system

**DOI:** 10.1038/s41443-023-00668-7

**Published:** 2023-02-13

**Authors:** Michela Fusaschi

**Affiliations:** University of Rome 3, Department of Political Science, Via G. Chiabrera, 199, 00145 Rome, Italy

**Keywords:** Health care, Quality of life, Human behaviour

Correction to: *International Journal of Impotence Research* 10.1038/s41443-022-00542-y, published online 04 March 2022

The article “Gendered genital modifications in critical anthropology: from discourses on FGM/C to new technologies in the sex/gender system”, written by Michela Fusaschi, was originally published electronically on the publisher’s internet portal on 04 March 2022 without open access. With the author(s)’ decision to opt for Open Choice the copyright of the article changed on 27 December 2022 to © The Authors 2022 and the article is forthwith distributed under a Creative Commons Attribution 4.0 International License, which permits use, sharing, adaptation, distribution and reproduction in any medium or format, as long as you give appropriate credit to the original author(s) and the source, provide a link to the Creative Commons licence, and indicate if changes were made. The images or other third party material in this article are included in the article’s Creative Commons licence, unless indicated otherwise in a credit line to the material. If material is not included in the article’s Creative Commons licence and your intended use is not permitted by statutory regulation or exceeds the permitted use, you will need to obtain permission directly from the copyright holder. To view a copy of this licence, visit http://creativecommons.org/licenses/by/4.0.

