# Supplementary material for: Gendered genital modifications in critical anthropology: from discourses on FGM/C to new technologies in the sex/gender system
Source: Int J Impot Res. 2022 Mar 4;35(1):6–15. doi: 10.1038/s41443-022-00542-y (PMC9935393; doi:10.1038/s41443-022-00542-y)
Supplement: Supplementary file 1 — Appendix 1 [file 41443_2022_542_MOESM1_ESM.pdf]

Selective chronology of FGM/C' s order of discourse

Pivotal moments/actors	Actions/proposal	Critical points and bibliographic suggestions
**1947**-The Executive Board of the American Anthropological Association (AAA) prepared a Statement on human rights and submitted it to the UN Commission on Human Rights.<br>(World Health Organization (WHO) as a specialized agency of the United Nations was established on 7 April 1948)<br><br>https://lucian.uchicago.edu/blogs/around1948/files/2012/09/1947-Statement-on-Human-Rights-American-Anthropological-Association.pdf	AAA critiqued the ethnocentric posture of the UN, supporting instead respect for difference and value pluralism through the concept of cultural relativism.	**Ethnocentrism, universalism, and cultural relativism.**<br><br>Colchester M. Cultural relativism and indigenous rights: Rethinking some dilemmas in applied anthropology. Anthropology Today. 2021; 37(3):16-19.<br>Dembour M. Following the movement of a pendulum: between universalism and relativism. In: Cowan JK, Dembour M, Wilson RA, editor. Culture and rights. 10 ed. Cambridge University Press; 2001. pag. 56-79.<br><br>Earp BD. Between moral relativism and moral hypocrisy: reframing the debate on "FGM". Kennedy Inst Ethics J. 2016; 26:105–44.<br><br>Merry SE. Human rights law and the demonization of culture (and anthropology along the way). Political and Legal Anthropology Review. 2003;26(1):55-76.
**1959**<br>United Nations Economic and Social Council resolution 680 BII (XXVI).<br>https://apps.who.int/iris/bitstream/handle/10665/87417/EB23R75_eng.pdf?sequence=1&isAllowed=y	WHO was invited to undertake a study "of the persistence of customs which subjects girls to ritual operations and of the measures adopted or planned for putting a stop to such practices" and communicate the inquire results to the Commission on the Status of Women. WHO, at first, had declined this invitation, stating that "the ritual operations in question are the result of social and cultural patterns which are outside the competence of the WHO".	**Start of 'anthropological vocabulary quarrels' and birth of the order of discourse.**
**1961** United Nations Economic and Social Council resolution 821 II (XXXII),<br>Resolutions adopted by the Economic and Social Council during its 32nd session,<br><br>https://digitallibrary.un.org/record/213494?ln=en#record-files-collapse-header	I Report of the Commission on the Status of Women. UN Economic and Social Council<br>again, invited WHO to study the medical aspects of genital operations based on 'custom'. Lack of response for almost a decade (1960s-1970s). The WHO and other international organisations, such as UNICEF, saw the FGM/C as a cultural problem (and not a public health one); they assumed that it needed to be analysed (e.g. data collection) and resolved by the local politics of the countries involved.	**Start of dislocation of meaning of the notions of culture, ritual operations and tradition: anthropological concepts were considered using an ahistorical and essentialist perspective.**<br><br>Boddy J. Womb as oasis: The symbolic context of pharaonic circumcision in rural northern Sudan. American Ethnologist. 1982;9(4):682-98.<br><br>Boddy JP. Civilizing Women: British Crusades in Colonial Sudan. Princeton. Princeton University Press; 2007.<br><br>Thomas L. 'Ngaitana (I will circumcise myself)': Lessons from Colonial Campaigns to Ban Excision in Meru, Kenya". In: Shell-Duncan B, Hernlund, Y editors, Female "Circumcision" in Africa. Lynne. 2000. p. 129-150.
**1975** World Conference of the International Women's Mexico City, Mexico<br><br>https://www.un.org/en/conferences/women/mexico-city1975	The first world conference on the status of women to coincide with the 1975 International Women's Year, observed to remind the international community that discrimination against women continued to be a persistent problem in much of the world.	**The Women's Decade (1975-1985) put "women" as a victimized category on the world's agenda.**<br><br>Tamale S. The right to culture and the culture of rights: a critical perspective on women's sexual rights in Africa. Feminist Legal Studies. 2008;16: 47–69.<br><br>Hayes RO. Female genital mutilation, fertility control, women's roles, and the patrilineage in modern Sudan: A functional analysis. American Ethnologist. 1975;2(4):617-33.<br><br>Oyěwùmí O. The invention of women. Making an African sense of Western gender discourses. NED-New edition. University of Minnesota Press; 1997.
**1979** Seminar on traditional practices affecting the health of women and children, Khartoum, organised by the WHO Regional Office for the Eastern Mediterranean in Khartoum<br><br>https://apps.who.int/iris/bitstream/handle/10665/254379/emmch137emment91emsemtrpraffhthwm44en.pdf?sequence=1&isAllowed=y	The seminar marked a milestone in the campaign against "harmful traditional practices", setting the pace and direction for international and national plans of action.<br>The third session was devoted to the discussion of 'female circumcision.'	**Tile third session was devoted to the discussion of 'female circumcision.'**<br>**Fran Hosken and the politicisation of the term Mutilation.**<br>**Female genital modifications were isolated graphically (Africa), defined and classified by four typologies (and ritual defloration and ritual dilatation completely disappeared). Male operations, such as circumcision, were expelled from "genital modifications" because not perceived to adversely affect health (apart from 'botched' operations).**<br>**North/South feminist debates. The FGM's definition as harmful traditional practices and the politics of naming.**<br><br>Earp BD, Darby R. Circumcision, autonomy and public health. Public Health Ethics. 2019;12(1):64-81. doi:10.1093/phe/phx024<br><br>Gosselin C. Feminism, anthropology and the politics of excision in Mali: Global and local debates in a postcolonial world. Anthropologica. 2000;42(1):43-60. doi:10.2307/25605957<br><br>Gruenbaum E. The Movement against Clitoridectomy and Infibulation in Sudan: Public Health Policy and the Women's Movement. Medical Anthropology Newsletter. 1982;13(2):4–12.<br><br>Longman C, Bradley T, editors. Interrogating harmful cultural practices: Gender, culture and coercion. Farnham, Surrey, England; Burlington, VT, Ashgate; 2015.<br><br>Mohanty CT. Under Western Eyes: Feminist Scholarship and Colonial Discourses. Feminist Review. 1988;(30):61.<br><br>Obermeyer CM. The health consequences of female circumcision: Science, advocacy, and standards of evidence. Medical Anthropology Quarterly. 2003;17(3):394-412.<br><br>Obermeyer CM. Female Genital Surgeries: The Known, the Unknown, and the Unknowable. Medical Anthropology Quarterly. 1999;13(1):79–106.<br><br>Oyěwùmí O, ed. Gender epistemologies in Africa: the gendering of African traditions, spaces, social institutions, and identities. New York: Palgrave Macmillan; 2011.<br><br>Walley CJ. Searching for Voices: Feminism, Anthropology, and the Global Debate over Female Genital Operations. Cultural Anthropology. 1997;12(3):405–38.

Pivotal moments/actors	Actions/proposal	Critical points and bibliographic suggestions
**1979** Adoption by the UN General Assembly of the Convention on the Elimination of All Forms of Discrimination against Women CEDAW<br><br>https://www.ohchr.org/documents/professionalinterest/cedaw.pdf	The art. 5 of the Convention demands to the UN's State parties to 'modify the social and cultural patterns of conduct of men and women, with a view to achieving the elimination of prejudices and customary and all other practices which are based on the idea of the inferiority or the superiority of either of the sexes or on stereotyped roles for men and women'.	**'FGM' vocabulary is problematic, cultural and gender essentialism.**<br><br>Bell K. Genital Cutting and Western Discourses on Sexuality. Medical Anthropology Quarterly. 2005;19(2):125-148.<br><br>Gosselin C. Feminism, anthropology and the politics of excision in Mali: Global and local debates in a postcolonial world. Anthropologica. 2000;42(1):43-60. doi:10.2307/25605957<br><br>Gruenbaum E. The Female Circumcision Controversy: An Anthropological Perspective. Philadelphia University of Pennsylvania Press; 2001.<br><br>Walley CJ. Searching for "Voices": Feminism, Anthropology, and the Global Debate over Female Genital Operations. Cultural Anthropology. 1997;12(3):405-438.<br><br>Obermeyer CM. Female Genital Surgeries: The Known, the Unknown, and the Unknowable. Medical Anthropology Quarterly. 1999;13(1):79-106.<br><br>Smith C. Who Defines "Mutilation"? Challenging Imperialism in the Discourse of Female Genital Cutting. Feminist Formations. 2011;23(1):25-46.
**1980** Second UN world conference on women in Copenhagen<br><br>(https://www.un.org/womenwatch/daw/beijing/otherconferences/Copenhagen/Copenhagen%20Full%20Optimized.pdf	Female circumcision" was a major topic of discussion in the NGO Forum, where organizations and activists mainly from the North advocated for the eradication of the practice.	**Female circumcision versus Female Genital Mutilation.**<br>**White feminism versus Black feminism.**<br>**The debate highlights the division between the global North women's movement and global South. The Western women revealed 'intellectual neocolonialism', 'latent racism,' and 'anti-Arab and anti-Islamic fervor'.**<br><br>Gilliam A. Women's Equality and National Liberation, Third World Women and the Politics of Feminism in CT. Mohanty, A. Russo and L. Torres (eds.), Bloomington and Indianapolis: Indiana University Press; 1991. P. 215-236.<br><br>Shell-Duncan B, Hernlund Y. editors. Female "circumcision" in Africa: Culture, controversy, and change. Boulder: Lynne Rienner Publishers; 2000. p. 283–315.
**1985** Third UN World Conference on Women in Nairobi<br><br>https://www.un.org/womenwatch/daw/beijing/otherconferences/Nairobi/Nairobi%20Full%20Optimized.pdf)	The concept of gender mainstreaming was first introduced.	**Gender mainstreaming and empowerment became the passe-partout of global hegemonic differentialist politics.**<br><br>Boyle EH. Female genital cutting: cultural conflict in the global community. Baltimore: Johns Hopkins University Press; 2002.<br><br>Fusaschi M, Cavatorta G. editors FGM/C: From medicine to critical anthropology. Torino: METI Edizioni; 2018.<br><br>Hernlund Y, Shell-Duncan B. editors Transcultural Bodies: Female Genital Cutting in Global Context. New Brunswick, N.J Rutgers University Press; 2007.<br><br>Hodžić S. Ascertaining deadly harms: aesthetics and politics of global evidence. Cult Anthropol. 2013;28:86–109.<br><br>Merry SE. Crossing Boundaries: Ethnography in the Twenty-First Century. Political and Legal Anthropology Review. 2000;23(2):127-133.
**1987** Regional Seminar on Traditional Practices Affecting the Health<br>of Women and Children in Africa, Addis' Ababa, Ethiopia organised by the Inter-African Committee on Traditional Practices Affecting the Health on Women and Children (IAC), in collaboration with the WHO, and co-sponsered by UNICEF.<br><br>https://apps.who.int/iris/bitstream/handle/10665/62930/24857eng.pdf?sequence=1&isAllowed=y	The expression "female circumcision" is used to indicate "the partial or complete removal of the female external genitalia".	**Further problems of terminology.**<br>**Female circumcision versus Female Genital Mutilation**<br><br>Johnsdotter S, Johansen, EB. Introduction. In: Johnsdotter S. (ed.) Female genital cutting: The global north and south. Holmbergs, Malmö . Malmö universitet; 2020. p.8–10.<br><br>Gosselin C. Feminism, anthropology and the politics of excision in Mali: Global and local debates in a postcolonial world. Anthropologica. 2000;42(1):43-60. doi:10.2307/25605957<br><br>Gruenbaum E. Tensions and movements: Female genital cutting in the global North and South, then and now. In: Johnsdotter S. (ed.) Female genital cutting: the global North and South. Holmbergs, Malmö. Malmö universitet; 2020. p.23–58.
**1990** CEDAW's General Recommendation No. 14 (Ninth session) on "Female circumcision"<br><br>https://www.un.org/womenwatch/daw/cedaw/recommendations/recomm.htm	The CEDAW recommends to State parties to "take appropriate and effective measures with a view to eradicate the practice of female circumcision". Among those measures there is: the support of women's local organizations, the introduction of training programmes, the encouragement of religious and community leaders at all level to cooperate at all levels.	**Circumcision versus mutilation and the 'imperfect glossaries' based on ahistorical notions**<br>**Problems of local impact and vernacularisation.**<br><br>Earp BD Female genital mutilation (FGM) and male circumcision: Should there be a separate ethical discourse? Practical Ethics. University of Oxford; 2014. Available from: https://philpapers.org/archive/EARFGM.pdf<br><br>Merli C. Male and female genital cutting among Southern Thailand's Muslims: rituals, biomedical practice, and local discourses. Culture, Health & Sexuality. 2010;12(7):725–38.<br><br>Merry SE. Human Rights and Gender Violence: Translating International Law into Local Justice. University of Chicago Press; 2006.<br><br>Johnsdotter S, Essén B. Genitals and ethnicity: The politics of genital modifications. Reprod Health Matters. 2010;18:29–37.<br><br>Shell-Duncan, B., Moreau, A., Smith, S., & Shakya, H. Women's business? A social network study of the influence of men on decision-making regarding female genital mutilation/cutting in Senegal. Global Public Health. 2021; 16(6): 856-869, DOI: 10.1080/17441692.2020.1826996

Pivotal moments/actors	Actions/proposal	Critical points and bibliographic suggestions
**1993** World Conference on Human Rights in Vienna<br><br>https://www.ohchr.org/en/aboutus/pages/viennawc.aspx	Declaration on the Elimination of Violence against Women and slogan introduction 'Women's rights are human rights!'<br><br>The World Conference on Human Rights stresses the importance of working towards the elimination of violence against women in public and private life, the elimination of all forms of sexual harassment, exploitation and trafficking in women, the elimination of gender bias in the administration of justice and the eradication of any conflicts which may arise between the rights of women and the harmful effects of certain traditional or customary practices, cultural prejudices and religious extremism.	**The paradigm change: from health to human rights: significant criticism by the post- and de-colonial feminist movement**<br><br>Asad T. What Do Human Rights Do? An Anthropological Enquiry. Theory & Event. 2000; 4(4): muse.jhu.edu/article/32601.<br><br>Gosselin C. Feminism, anthropology and the politics of excision in Mali: Global and local debates in a postcolonial world. Anthropologica. 2000;42(1):43-60. doi:10.2307/25605957<br><br>Shell-Duncan B. From health to human rights: Female genital cutting and the politics of intervention. American Anthropologist. 2008;110(2):225-36.
**1994** International Conference of Parliamentararians on Population and Development in Cairo, organized by the United Nations Population Found (UNFPA)<br><br>https://www.unfpa.org/resources/cairo-declaration-population-development	At the paragraph entitled *The girl child* (under the chapter *Gender Equality, Equity and Empowerment of Women*). The objectives are to eliminate all forms of discrimination against the girl child, to eliminate the root causes of son preference, to increase public awareness of the value of the girl child and to strengthen her self-esteem.<br>Governments and communities are advised to take steps urgently to stop the practice of female genital mutilation and protect women and girls from all similar unnecessary and dangerous practices. "Governments are urged to prohibit female genital mutilation wherever it exists".	**Gender equality and equity and the empowerment of women, the elimination of all kinds of violence against women.**<br><br>Ahmadu FS. Rites and wrongs: An insider/outsider reflects on power and excision. In: Shell-Duncan B, Hernlund Y. (eds.) Female "circumcision" in Africa: Culture, controversy, and change. Boulder: Lynne Rienner Publishers; 2000. p. 283-315.<br><br>Ahmadu FS. Shweder Richard A Disputing the myth of the sexual dysfunction of circumcised women: An interview with Fuambai S. Ahmadu by Richard A. Shweder". Anthropology Today.2009 25 (6): 14-17.<br><br>Merry SE. Gender violence: a cultural perspective. Oxford Wiley-Blackwell Pub; 2009.
**1995** 4th UN World Conference on Women in Beijing<br><br>https://www.un.org/en/conferences/women/beijing1995	Platform for Action calls for the elimination of violence against women, which is a human rights violation, resulting from harmful traditional or customary practices, cultural prejudices and extremism	**Definition of the order of discourse and adoption of glossary at the global level.**<br><br>Earp BD, Darby R. Circumcision, autonomy and public health. Public Health Ethics. 2019;12(1):64–81. doi:10.1093/phe/phx024<br><br>Grande E. Hegemonic human rights: the case of female circumcision. A call for taking multiculturalism seriously. Arch Antropol Mediterr. 2009; 12:11–27.<br><br>Johnsdotter S. Meaning well while doing harm: compulsory genital examinations in Swedish African girls. Sexual and Reproductive Health Matters. 2019. 27(2):87–99.<br><br>Logie CH, Perez-Brumer A, Parker R. The contested global politics of pleasure and danger: Sexuality, gender, health and human rights. Glob Public Health. 2021 May;16(5):651-663. doi: 10.1080/17441692.2021.1893373. PMID: 33904384.
**2003** International Conference on "Zero tolerance to FGM", organized by the INTER-AFRICAN COMMITTEE (IAC)<br>On traditional practices affecting women's and children's health<br>Addis Ababa, Ethiopia<br><br>https://www.help-africanwomen.org/images/downloads/FGM-Themen/FGM-Null_Toleranz-EN-web.pdf	February 6 has been designated by the United Nations as the International Day of Zero Tolerance for Female Genital Mutilation (FGM)	**Zero tolerance for FGM approach.**<br><br>Boddy J. Re-thinking the Zero Tolerance Approach to FGM/C: the Debate Around Female Genital Cosmetic Surgery. Curr Sex Health Rep. 2020;12(4):302-313. doi:10.1007/s11930-020-00293-1.<br><br>Londoño Sulkin CD Anthropology, liberalism and female genital cutting. Anthropology today. 2009;25(6): 17–19. doi:10.1111/j.1467-8322.2009.00700.x<br><br>Earp BD, Johnsdotter S. Current critiques of the WHO policy on female genital mutilation. Int J Impot Res. 2021;33(2):196–209.<br><br>O'Neill S, Bader D, Kraus C, Godin I, Abdulcadir J, Alexander S. Rethinking the Anti-FGM Zero-Tolerance Policy: from Intellectual Concerns to Empirical Challenges. Curr Sex Health Rep. 2020;12(4):266–75.
**2004**. U.S. Agency for International Development (USAID). 2000. *USAID policy on female genital cutting (FGC)*. Washington, D.C.: USAID.<br><br>https://dhsprogram.com/pubs/pdf/cr7/cr7.pdf<br><br>See also UNFPA. (2019). Evaluation of the UNFPA-UNICEF joint programme on the abandonment of female genital mutilation: Accelerating change.<br><br>https://www.unfpa.org/admin-resource/joint-evaluation-unfpa-unicef-joint-programme-abandonment-female-genital-mutilation.	DHS Comparative Reports: Female Genital Cutting in the Demographic and Health Surveys:<br>A Critical and Comparative Analysis: this comparative report summarizes data on FGC from Demographic and Health Surveys (DHS) implemented between 1989 and 2002.<br><br>The more neutral term, *female genital cutting*, preferred by an increasing number of researchers, is the term recommended by the United States Agency for International Development (USAID)	**Terminologies, seduction of quantification and moral economies analysis.**<br><br>Andro A, Lesclingand M, Grieve M, Reeve P. Female genital mutilation. Overview and current knowledge. Population. 2016;71:215–96.<br><br>Cappa C, Van Baelen L, Leye E. The practice of female genital mutilation across the world: Data availability and approaches to measurement. Glob Public Health. 2019;14(8):1139–52.<br><br>Elamin W, Mason-Jones AJ. Female genital mutilation/cutting: A systematic review and meta-ethnography exploring women's views of why it exists and persists. International journal of sexual health. 2020;32(1):1-21.<br><br>Fusaschi M. Humanitarian bodies: Gender, moral economy and genital modification in Italian immigration policy. Cahiers d'Études Africaines. 2015;217(1):11–28. doi:10.4000/etudesafricaines.17985<br><br>Merry SE, Davis KE, Kingsbury B, eds. The quiet power of indicators: measuring governance, corruption, and the rule of law. New York, NY: Cambridge University Press; 2015.<br><br>Merry SE. The seductions of quantification: measuring human rights, gender violence, and sex trafficking. Chicago: The University of Chicago Press; 2016. p. 249<br><br>Johnsdotter S. (ed.) Female genital cutting: the global North and South. Holmbergs, Malmö. Malmö universitet; 2020.<br><br>Shell-Duncan B, Njue C, Moore Z. Trends in medicalisation of female genital mutilation/cutting: What do the data reveal? [Internet]. Population Council. Available at: https://knowledgecommons.popcouncil.org/departmentssbsr-rh/571

Pivotal moments/actors	Actions/proposal	Critical points and bibliographic suggestions
**2008** Eliminating female genital mutilation. An interagency statement - OHCHR, UNAIDS, UNDP, UNECA, UNESCO, UNFPA, UNHCR, UNICEF, UNIFEM, WHO<br><br>https://apps.who.int/iris/bitstream/handle/10665/43839/9789241596442_eng.pdf?sequence=1	This Statement is a call to all States, international and national organizations, civil society and communities to uphold the rights of girls and women. It also calls on those bodies and communities to develop, strengthen, and support specific and concrete actions directed towards ending female genital mutilation	**New classification, Type IV in the annexes?** (See Box 2)<br><br>Fusaschi M. A paradoxical Rwandan female genital "mutilation". In Fusaschi M, Cavatorta G, (eds). *FGM/C:* From medicine to critical anthropology. Rome: Meti; 2018, p. 107-23.<br><br>Fusaschi M. Trouble dans le gukuna rwandais: Fémocratie, féminismes et anthropologie critique. Anuac. 2020;9(2):17–43. doi:10.7340/ANUAC2239-625X-4102.<br><br>Longman C, Bradley T, editors. Interrogating harmful cultural practices: Gender, culture and coercion. Farnham, Surrey, England; Burlington, VT, Ashgate; 2015.
**2009** UN report to the General Assembly on the Girl Child<br><br>https://www.refworld.org/docid/4ac9ac552.html	Section Efforts to support the abandonment of female genital mutilation/cutting: 'FGM is "perpetrated without a primary intention of violence but is de facto violent in nature' (p. 16)	**The concept of violence is extremely unstable.**<br><br>Das V. The Anthropology of violence and the speech of victims. Anthropology Today. 1987;3(4):11–3.<br><br>Das V, Kleinman A, Ramphele M., Reynolds P editors. Violence & Subjectivity, Berkeley: University of California Press; 2000.
**2011** Council of Europe Convention on Preventing and Combating Violence against Women and Domestic Violence (the Istanbul Convention)<br><br>https://rm.coe.int/168008482e	The Istanbul Convention requests that State Parities criminalise FGM	**The impact of FGM criminalisation.**<br><br>Florquin S, Richard F. Critical discussion on female genital cutting/mutilation and other genital alterations: Perspectives from a women's rights NGO. Curr Sex Health Rep. 2020;12(4):292-301. doi:10.1007/s11930-020-00277-1.<br><br>Fusaschi M. Quel genre de convictions dans les Conventions ? Esquisses d'auto-ethnographie des droits humains des femmes en tant qu'économies morales : le cas des Modifications Génitales Féminines. AAM. 2020; 22(1). Available at: http://journals.openedition.org/aam/2832.<br><br>Peroni L. Violence Against Migrant Women: The Istanbul Convention Through a Postcolonial Feminist Lens. Fem Leg Stud. 2016;24(1):49–67.
**2012** UN General Assembly Resolution *Intensifying Global Efforts for the Elimination of Female Genital Mutilations*<br><br>https://undocs.org/pdf?symbol=en/A/RES/67/146	The resolution, 'Intensifying Global Efforts for the Elimination of Female Genital Mutilations', calling on all countries to enact legislation banning FGM, as well as raising awareness and allocating sufficient resources to protect and support women and girls	**Criminal Legislation is questionable.**<br><br>Antonazzo M. Problems with Criminalizing Female Genital Cutting. Peace Review. 2003;15(4):471–7. doi: 10.1080/1040265032000156663 Bader D Mottier V. Femonationalism and populist politics: The case of the Swiss ban on female genital mutilation. Nations and Nationalism, 2020; 26 (3): 644-659<br>Cloward K. False Commitments: Local Misrepresentation and the International Norms Against Female Genital Mutilation and Early Marriage. International Organization. 2014;68(3):495–526.<br><br>Earp BD, Johnsdotter S. Current critiques of the WHO policy on female genital mutilation. Int J Impot Res. 2021;33(2):196–209.<br><br>Merry SE. Human Rights and Gender Violence: Translating International Law into Local Justice. University of Chicago Press; 2006.
**2012** European Parliament resolution of 14 June 2012 on ending female genital mutilation (2012/2684(RSP)<br><br>https://www.europarl.europa.eu/doceo/document/TA-7-2012-0261_EN.html	States that the data of women and girls living with FGM or at risk are 'underestimated and do not take into account second-generation or undocumented migrants'.	**Migration context as a new challenge to understand the phenomenon FGM/C in the EU.**<br><br>Gele AA, Kumar B, Hjelde KH, Sundby J. Attitudes toward female circumcision among Somali immigrants in Oslo: A qualitative study. International Journal of Women's Health. 2012;4:7-17.<br><br>O'Neill S, Richard F, Vanderhoven C, Caillet M Pleasure, womanhood and the desire for reconstructive surgery after female genital cutting in Belgium: Anthropology & Medicine. 2021; DOI: 10.1080/13648470.2021.1994332<br><br>Johansen RE. Undoing female genital cutting: Perceptions and experiences of infibulation, defibulation and virginity among Somali and Sudanese migrants in Norway. Culture, Health & Sexuality. 2017;19(4):528-42.<br><br>Johnsdotter, S., Essén, B. Deinfibulation Contextualized: Delicacies of Shared Decision-Making in the Clinic. Arch Sex Behav 2021; 50**:** 1943–1948 https://doi.org/10.1007/s10508-020-01676-0
